# A Re-Examination of the History of Etiologic Confusion between Dengue and Chikungunya

**DOI:** 10.1371/journal.pntd.0004101

**Published:** 2015-11-12

**Authors:** Goro Kuno

**Affiliations:** Retired, Fort Collins, Colorado, United States of America; Pediatric Dengue Vaccine Initiative, UNITED STATES

## Abstract

Contrary to the perception of many researchers that the recent invasion of chikungunya (CHIK) in the Western Hemisphere marked the first episode in history, a recent publication reminded them that CHIK had prevailed in the West Indies and southern regions of the United States from 1827–1828 under the guise of “dengue” (DEN), and that many old outbreaks of so-called “dengue” actually represented the CHIK cases erroneously identified as “dengue.” In hindsight, this confusion was unavoidable, given that the syndromes of the two diseases—transmitted by the same mosquito vector in urban areas—are very similar, and that specific laboratory-based diagnostic techniques for these diseases did not exist prior to 1940. While past reviewers reclassified problematic “dengue” outbreaks as CHIK, primarily based on manifestation of arthralgia as a marker of CHIK, they neither identified the root cause of the alleged misdiagnosis nor did they elaborate on the negative consequences derived from it. This article presents a reconstructed history of the genesis of the clinical definition of dengue by emphasizing problems with the definition, subsequent confusion with CHIK, and the ways in which physicians dealt with the variation in dengue-like (“dengue”) syndromes. Then, the article identifies in those records several factors complicating reclassification, based on current practice and standards. These factors include terms used for characterizing joint problems, style of documenting outbreak data, frequency of manifestation of arthralgia, possible involvement of more than one agent, and occurrence of the principal vector. The analysis of those factors reveals that while some of the old “dengue” outbreaks, including the 1827–1828 outbreaks in the Americas, are compatible with CHIK, similar reclassification of other “dengue” outbreaks to CHIK is difficult because of a combination of the absence of pathognomonic syndrome in these diseases and conflicting background information.

## Introduction

In recent decades, we have witnessed dramatic increases in the frequency and magnitude of chikungunya (CHIK) in dengue-endemic regions in Africa, Asia, and the Pacific; beginning in late 2013, CHIK suddenly emerged in the Western Hemisphere.

To the surprise of investigators who thought that the latest CHIK outbreak was the first episode in the Americas, a recent article reminded them that CHIK outbreaks had occurred in the West Indies and southern states of the United States in 1827–1828, and that many outbreaks of so-called “dengue” (DEN) recorded before the advent of laboratory-based confirmatory techniques (which began to evolve in the late 1940s) represented cases of mistakenly identified CHIK [1]. Actually, this problem was first discovered by James Christie in 1881 [2]. Much later, Donald E. Carey’s re-examination—largely based on the list of “dengue” outbreaks previously reviewed by Christie—reclassified some of them as CHIK outbreaks [3]. Although Carey’s contribution has been occasionally cited in medical literature [4,5], only two investigators [1,6] independently re-examined the original references used by Carey. Furthermore, no one has attempted to identify the root of the etiologic confusion, which persisted for nearly 150 years. It is difficult to assess the quality or accuracy of Carey’s CHIK reclassification without the provision of historical background information, analyses of the negative impacts of the definition, consideration of the peculiarities of outbreak documentation in old records, or scrutiny of other factors (such as entomologic data and other possible etiologic agents).

Accordingly, this article first presents a reconstructed history of dengue definition, its complication with CHIK, and other negative impacts. Then, it identifies the factors complicating retrospective reclassification of old “dengue” to CHIK.

## Methods

The major sources of early DEN and CHIK documents used for this review are comprehensive treatises of early “dengue” outbreaks compiled by August Hirsch and George Melvyn Howe [7,8] and a bibliographic database [9]. In the text, the word “dengue” in double quotation marks is used in reference to the etiology of dengue-like illness or outbreaks not definitively confirmed by the current standards. The same practice is applied to CHIK and O’nyong nyong fever (ONN) as well. This analysis of the factors complicating reclassification is based only on the “dengue” outbreaks selected in Carey’s review [3].

## History of the Clinical Definition of Dengue-Like Illnesses in Relation to Misdiagnosis with Chikungunya

Establishing a clinical definition of dengue was known to be notoriously difficult in tropical environments because of the existence of multiple infectious diseases manifesting similar syndromes. Although this article is about misdiagnosis with CHIK, the confusion between “dengue” and other viral diseases ranges from a myth that it is a benign form of yellow fever to a belief that it is a tropical variety of influenza. The traditional definition also fostered a practice of lumping multiple diseases known under various names in different parts of the tropics into one category, such as “dengue.” The reconstructed history below reveals how the dengue definition originated, the problems that emerged, how physicians dealt with the variations of the syndrome, and how CHIK entered into the history of misdiagnosis.

### 1658–1801

The earliest record of the occurrence of severe arthralgia (observed in Cairo, Egypt in 1658), which was retrospectively found to be more compatible with CHIK much later, was noted by Melchisédec de Thévenot in his book *Relations de divers voyages curieux qui nont point este publiees* (Paris: Thomas Mortte; 1672; partial translation in English by Da Cunha) [10].

According to Spanish archives, the words “quebranta huesos” (break bone syndrome) were used by physicians in Puerto Rico as early as 1771, and the word “dengue” was used in Spain in 1801 with respect to an unidentified condition [11].

### 1779–1780: “Knokkel-koorts” in Batavia (now Jakarta, Indonesia) and “break bone fever” in Philadelphia

The outbreak described by David Bylon in Batavia [12] is highly compatible with CHIK and agrees with Carey’s reclassification [3].

As for the 1780 outbreak in Philadelphia, it has traditionally been considered the first “dengue” epidemic in North America. Although Carey also classified it as “dengue” [3], to identify the etiology of “bilious remitting fever,” described by Benjamin Rush [13], is actually very difficult. Rush’s brief mention of some patients suffering from severe pains in joints may even be compatible with CHIK. However, his style of extensively recording the variation of symptoms, ranging from gastrointestinal problems to manifestation of jaundice, renders etiologic interpretation difficult. James G. Thomas remarked that Rush probably confused the outbreak with yellow fever (YF) [14].

The origin of the term “break bone fever” (which Rush believed to be a form of “rheumatism”) remains unknown, but at least the term was not Rush’s coinage. Thomas dismissed the term because it had commonly been used, nonspecifically, by laypersons in the country to express a variety of bodily pains [14]. Later, the “break bone” symptom was sporadically described in reports but generally found to occur rather infrequently in “dengue” [15,16], and the term fell into disuse.

### 1824–1825: Outbreaks in India

According to Christie, an epidemic of “kidinga pepo” occurred in East Africa from 1823–1824 and then spread to India, resulting in extensive outbreaks [2]. Because of the manifestation of severe arthralgia in the Indian outbreak [17], suspicion of CHIK is strong, as interpreted by Carey [3]. However, this is puzzling because, according to a theory, importation of the major CHIK vector, *Aedes aegypti*, to tropical Asia (including India) occurred only in the second half of the 19th century [18]. If the etiology of this outbreak was CHIK, transmission by an Asian mosquito (*Ae*. *albopictus*) cannot be ruled out. Also, the report of many cases of “relapse” within the outbreak [17] is of interest. In contrast to the traditional usage of this term—to mean the return of the same symptom within one episode of infection—some cases of “relapse” in this report strongly suggest more than one experience of the same disease or similar diseases within one epidemic period. This raises a question about CHIK-only etiology because reinfection with Chikungunya virus (CHIKV) is unlikely. On the other hand, if the true etiology was DEN, these cases could be explained by the involvement of multiple Dengue virus (DENV) serotypes.

### 1826–1828: “Dengue” outbreaks in the West Indies and southern states of the US

“Dengue” outbreaks, which erupted mostly in 1827 in the West Indies, spread to the southern states of the US. The word “dengue” began to be used popularly among Spanish-speaking people during an outbreak in Havana, Cuba [19]. As interpreted by Carey, the likelihood of the CHIK or CHIK-like illness in this pandemic is very strong, given so many reports of severe arthralgia [19–23]. W.H. Ruan [23] clearly recognized that this outbreak was distinct from the “break bone fever” of 1780 that Rush described. Regarding arthralgia, a closer examination of other documents from the Caribbean revealed either no reference to it or, if reported, only mild cases of short duration [24]. When hospital admission due to rheumatism in the British West Indian garrisons is used as a proxy indicator of an epidemic of a disease resembling CHIK, the data in the period covering 1827–1828 do not reveal any significant spike, but the rate of “eruptive fever” was higher during this period (several years) as compared to those in the periods before and after [25].

### 1869: Joint Committee of the Royal College of Physicians, London

In an attempt to systematically classify human diseases, a consortium of medical organizations serving the Crown—the Joint Committee of the Royal College of Physicians (hereafter called the Joint Committee)—compared various systems proposed by nosologists in Europe and spent ten years coming up with a reasonable scheme. The head of the Joint Committee was William Aitken, an authority on infectious diseases who, in 1866, had defined “dengue” as “a febrile affection, sui generis, commencing suddenly, associated from the commencement with severe joint pains in the large and small joints…” [26]. His emphasis on arthralgia likely reflected his extensive reading of the reports from the West Indies during the 1827–1828 outbreaks.

The Joint Committee classified 1,146 diseases by a system principally designed by William Farr. “Dengue” was assigned to “general diseases,” with a Latin name (“denguis”). The Joint Committee allocated only one name, “dengue,” for all similar diseases known by different names. “Dengue” was defined as “an ephemeral continued fever, characterised by frontal headache, and by severe pains in the limbs and trunks, and sometimes by an eruption resembling that of measles over the body; occurring in the West Indies” [27]. All “dengue” definitions at the time were understood in the unstated context of the abrupt emergence of an outbreak of febrile, exanthematous, and usually nonfatal illness affecting a large portion of people in urban areas with warm climates. The clinical syndrome of “dengue” used for this definition derived mostly from the observations in the 1827–1828 outbreaks in the West Indies. This reflected the interest, already noted, of the head of the Committee. If Carey’s interpretation of misdiagnosis of CHIK as “dengue” in the 1827–1828 West Indies outbreak [3] is correct, etiologic confusion must have originated in this definition by the Joint Committee. Shortly thereafter, however, this definition was criticized because it was found incompatible with the syndrome observed in India in 1872 [28].

### 1871–1872: “Kidinga pepo” in Zanzibar, Tanzania

James Christie observed an outbreak of a febrile, exanthematous illness characterized by pronounced arthralgia in Zanzibar, Tanzania in 1870–1871, and he reported the disease as “kidinga pepo,” a term in the local Swahili language that expresses the characteristic joint pain [29]. After consulting with William Aitken, Christie learned of the “dengue” syndrome in the West Indies during the 1827–1828 outbreaks and recognized the similarity with “kidinga pepo.” The etiology of “kidinga pepo” [29] in Zanzibar is highly compatible with CHIK, as interpreted by Carey [3]. But, strictly speaking, it remains unknown because of a possibility of another arthralgic disease, ONN—meaning severe joint pain in the language of the Acholi tribe in northern Uganda—which is indigenous in West, Central, and East Africa.

### 1872: India, China

The pandemic, which apparently originated in East Africa in 1871, hit India hard in 1872. Recognizing the severity of arthralgia, Joseph G. Da Cunha, then in Bombay (now Mumbai), essentially concurred with Christie that “kidinga pepo” was distinct from “dengue” in several particulars when the disease syndrome was compared with the traditional characterization by the East Indian physicians [10].

Based just on severe and persistent arthralgia, the clinical syndrome reported in Calcutta (now Kolkata) is compatible with CHIK [28,30]. The physicians there, however, were puzzled by a variation in “dengue” symptoms and speculated that “dengue” was “a compound of two diseases—that it was caused by a mixture of two kinds of materies morbi, one of which is like that of eruptive fever, and the other the same as rheumatism or gout” [14]. This was not the only diagnostic complication. A contemporary report from Pondicherry (then a French colony and now called Puducherry) described an unusual syndrome manifesting cardiovascular dysfunctions, central nervous system (CNS) syndrome, and coma, followed by death. Of the 25 fatal cases, 17 were children [31]. This syndrome was quite distinct from the “dengue” reported elsewhere in India at the time. The repeated mention of “typhoid condition” (meaning, at the time, stupor or confusion) preceding coma in the children in this report denotes a condition similar to shock. If they represented what is now known as dengue shock syndrome [32], the Indian cases would predate the first observation of such a severe “dengue” syndrome (recorded by Francis Everard Hare in Queensland, Australia in 1897) [15].

This pandemic spread to southern China, where, in 1872, Patrick Manson witnessed the outbreak characterized by severe and persistent arthralgia. His observations of the disease constituted the backbone of the “dengue” chapter in his classic *Manson’s Tropical Diseases* [33]. If this represented dispersal of the Indian “CHIK” outbreak, obviously Manson’s use of the word “dengue” would have been inappropriate. Because Manson’s textbook was an authoritative source of tropical diseases for many years, the negative impact of his “dengue” chapter must not have been trivial. Also, in 1880, G. Mahé included “kidinga pepo” in his medical dictionary as one of nearly 50 synonyms of “dengue” known worldwide [34].

### 1881: Christie’s review

After returning to Scotland, Christie examined in depth many previous records of “dengue” outbreaks in the literature and recognized not only the distinction of “kidinga pepo” from “dengue” recorded in some but also found a strong compatibility with other “dengue” outbreaks—in particular, the pandemics in 1823–1829 and 1870–1875 (in East Africa, Asia, and Oceania) and 1827–1828 (in the West Indies and southern states of the US) [2]. He also determined the incompatibility of “kidinga pepo” with Rush’s “break bone fever” [13] but compatibility with Bylon’s “knokkel-koorts” [12]. Furthermore, Christie speculated that a hybridization of the germs of the two diseases explained the emergence of atypical “dengue” with unusually severe and persistent arthralgia [2].

### 1883: Hirsch’s monograph

August Hirsch, a professor of geographic medicine at the University of Berlin, compiled a large number of outbreak records of “dengue” and diseases with similar symptoms but different names (including “kidinga pepo” by Christie [29]) and organized the records in chronologic order and by geographic location in order to better understand the geographic dispersal of “dengue” [7]. Because he published the monograph under one disease name, “dengue,” the clinical syndrome understood by Hirsch was most likely a composite of multiple similar diseases. Accordingly, “kidinga pepo” of Christie was interpreted by Hirsch as one of the variations of “dengue” syndrome.

### 1905–1912: “Seven-day fever” in India

The exact etiology of the outbreaks called “seven-day fever” in parts of India, which was later classified as “dengue” by Carey [3], became a topic of enormous controversy there [35–37]. In 1907, Leonard Rogers consulted with Patrick Manson and jointly concluded that it was not “dengue.” Stocker concluded that it was a bacterial disease; much later, it was classified as leptospirosis [33].

### 1917–1948: “Dengue” in Africa

Numerous outbreaks of “dengue-like illness” characterized by severe arthralgia were reported in this period from Central and West Africa—in particular, in Burkina Faso, Ghana, Mali, Nigeria, Senegal, and Zaire [38–41]. Because one feature was severe joint pain, the etiologies of these outbreaks were more likely to be CHIK or ONN than DEN. After reviewing the clinical variation among many “dengue-like illnesses” reported in Ghana and Nigeria, G. M. Findlay and R. W. Brookfield concluded that more than one type of “dengue-like illness” existed in West Africa, under different names [39]. The syndrome observed in outbreaks of “dengue” in Dakar, Senegal in 1932 [41] and Coquilhatville in the Belgian Congo (now Mbandaka, Zaire) in 1948 [40] is considered compatible with CHIK or ONN due to the severity and persistence of arthralgia in many of the patients examined.

### 1953: Discovery of chikungunya in Tanzania

An outbreak of a febrile, exanthematous illness, characterized by severe arthralgia and prolonged joint pain, occurred in southern Tanzania in 1952–1953 [42–44]. The investigators, unaware of the earlier report of “kidinga pepo” by Christie [29]—and, puzzlingly, failing to recognize the significance of severe and persistent arthralgia observed in many patients examined in “dengue” outbreaks reported earlier from Senegal and the Belgian Congo (despite citing one of the two reports) [40,41]—named the new disease “chikungunya,” a term from the local language of the Makonde region of the country [43]. Had William Lumsden, Marion Robinson, and R. Ross read the publications of Christie and other records in Central and West Africa carefully, the word “chikungunya” would not have entered the lexicon of tropical medicine or virology.

### 1971: Carey’s review

During concurrent epidemics of DEN and CHIK in India in 1964, Donald Edward Carey, then a physician at the Rockefeller Foundation, clearly recognized the difference between the two diseases, primarily through the observation of severe and often persistent arthralgia in CHIK [45]. Upon examination of many early “dengue” records, he discovered Christie’s contribution and determined the compatibility of many old “dengue” outbreaks, and “kidinga pepo,” with true CHIK.

## Factors Complicating the Accuracy of Clinical Diagnosis in Relation to “Dengue” or “Chikungunya” Misdiagnosis

Despite the troubled history of the clinical definition of dengue, retrospective serology on the survivors of “dengue” human experiments or outbreaks (such as those in the Philippines from 1924–1930, South Africa in 1927, and Greece from 1927–1928) confirmed the accuracy of the clinical diagnosis in the early part of the 20th century. In this period, the correct identification of dengue-infected patients in epidemiologic studies and of viremic patients (in order to secure the source of the infectious material for human experiments or virus isolation) was similarly based on the “traditional,” if troubled, clinical definition of “dengue.”

Accordingly, to more clearly analyze the two contrasting consequences of the application of the old “dengue” definition, it is helpful to focus on the following factors that complicate retrospective reclassification studies.

### Arthralgia (or polyarthralgia)

Despite being aware of arthralgic Mayaro fever, Carey selected this symptom at the time of his review because he thought that, “so far as is known, CHIK is the only arthropod-borne virus that bestows upon its victims the severe, residual, articular pains so characteristic of all of the foregoing epidemics” [3]. This is important, because the quality of Carey’s interpretation of early CHIK epidemics partly hinges on the accuracy of his assumption.

According to a modern definition by Joe G. Hardin, which was adopted by the National Institutes of Health of the US, “arthralgia” is segregated from “arthritis” because of the absence of inflammation; “arthritis” refers to the causal mechanism but is not a symptom itself [46]. In infectious disease reporting, however, such a clear distinction between arthralgia and arthritis has not been universally practiced or accepted. For reporting joint pain, depending on authors, terms such as “rheumatism” and “arthritis” have been used almost interchangeably with “arthralgia,” and this still occurs [47,48]. Therefore, using the modern definitions of terms that pertain to joint problems is often problematic in retrospective studies.

### Peculiarities of epidemiologic study and reporting in the old literature

Another problem in re-analyzing old “dengue” outbreaks is that community- or patient-based investigations that emphasized statistical significance and applied a case definition were not performed. Authors seldom recorded the total number of patients examined, how patients were selected, their ages or any underlying chronic illnesses, the frequency of the manifestations of a particular symptom or sign, or the duration of arthralgia. Accordingly, for the small number of cases of severe arthralgia that were recorded, it is difficult to determine whether they represented all (and the only) atypical cases worth documenting among a large number of patients examined or just a sample from many patients with the same symptom.

### Manifestation of arthralgia in CHIK and DEN

The high frequency of arthralgia in symptomatic CHIK patients, reported as high as 96.6% [48], is clearly a prominent feature that should be useful in differential diagnosis. However, the sampling of available data below indicates limitations due to its variability. In one report, severe arthralgia developed in 40% of CHIK- and 12% of DEN-hospitalized children [49]. As for adults, in a study of hospitalized cases of dengue hemorrhagic fever, 52.3% of the patients experienced arthralgia [50]. Although development of persistent (>3 months after onset of illness) arthralgia is clearly far less frequent in DEN than in CHIK, the frequency varies depending on the location, time, age, and underlying chronic condition of the patients. As an example, among confirmed DEN cases, arthralgia persisted for longer than six months in 10.7% of the cases, while, in another report, 57% of patients with underlying chronic illness (such as diabetes, gout, rheumatism, or other forms of arthritis) experienced persistent arthralgia for as long as two years [51,52]. Conversely, arthralgia in CHIK may be absent or benign. As an example, the duration of arthralgia in most patients in one laboratory-confirmed study was less than two weeks, with the longest being only five weeks [53]. In a more recent report on the CHIK patients with persistent joint problems (“rheumatic disorders”), 95% of them had musculoskeletal problems but not polyarthritis, while only 5% met the criteria for polyarthritis [47]. Regarding diagnostic variation among physicians, L. C. Scott surveyed diagnoses of bone and joint pains during a dengue outbreak in 1922 by sending a questionnaire to 2,000 physicians. Nearly 85% of the physicians characterized the symptoms as musculoskeletal pains around (but not in) joints, while only 15% confined pains exclusively to joints [54].

### Arboviruses other than CHIKV known for arthralgia and possible concurrent (or consecutive) outbreaks

By the time of Carey’s review, 1971, the other arboviruses known for severe arthralgia (such as *Mayaro*, ONN, *Ross River*, and *Sindbis*) had been isolated, except for *Barmah Forest* virus. Furthermore, an ONN outbreak in Africa (including Tanzania) from 1959–1963 had resulted in more than 2 million patients [55,56]. But, at the time, it was not well-recognized that persistent arthralgia also develops in DEN, although at a lower frequency than CHIK.

The possibility of concurrent or consecutive outbreaks by DENV and CHIKV, or by multiple serotypes of DENV, is raised by the increased tendency of manifestation of severe arthralgia in severe cases of DEN, as mentioned elsewhere in this article. Concurrent outbreaks are hinted at in the 1824 epidemic in India because of multiple references to episodes of “relapse” during the outbreaks [17]. The true etiologies of the 1826–1827 outbreaks in Charleston, South Carolina and Savannah, Georgia and the 1827–1828 outbreak in Charleston were identified by Carey [3] as “DEN” and “CHIK,” respectively. Because of temporal overlap, Carey’s interpretation strongly suggests either concurrent or consecutive outbreaks of the two diseases, at least in Charleston. Assuming that the true etiology of the 1872 “dengue” outbreak in India was CHIK (according to Carey [3]), the severe and unusual syndrome (atypical of CHIK) observed in children (including shock-like symptoms), coupled with high mortality in the age group [31], suggests that, at least in parts of India, DEN also occurred, because the development of shock in children is incompatible with CHIK [49]. According to the accounts of the Calcutta (now Kolkata) residents who experienced this outbreak, the severity of the symptoms changed in the course of the epidemic [14], suggesting a possibility of consecutive or concurrent outbreaks of CHIK and DEN.

## Closing Comments

A combination of the lack of pathognomonic syndrome and the unavailability of specific diagnostic methods in the past frequently contributes to the difficulty of retrospective etiologic identification. Furthermore, the meaning of a medical term pertaining to a particular symptom (or syndrome) or concept of a disease can shift over time, as shown in the example of pleurisy [57]. The definition for characterizing “joint pain” is another example—sources of the term have been variable and changing. According to one of the medical dictionaries in the 19th century, joint pain is covered in three terms: “arthralgia” (meaning joint pain and used synonymously with arthrodynia), arthritis (joint pain in gout and acute rheumatism), and arthrosia (joint pain with inflammation) [58]. However, the term “arthralgia” was not used in “dengue” reports in the 19th century. In another dictionary in the early 20th century, arthralgia was defined as “articular pain without appreciable lesion of the joint” [59]. Thus, because of the variation of definition and change in usage by physicians, caution is necessary in evaluating the etiologies of old “dengue” that are heavily based on arthralgia. While some of them, including the 1827–1828 outbreaks in the Americas, are compatible with CHIK, similar reclassification of other “dengue” outbreaks to CHIK is difficult because of a combination of the variability in reporting and conflicting background information. The critical review of old outbreaks also suggests intriguing possibilities about the development of the earliest epidemics of severe dengue and their spread.

**Key Learning Points**

1. Old biomedical literature should be regarded as another valuable resource in contemporary research rather than as a domain useful only for historians.
2. The limitations of differential diagnosis based only on clinical syndrome and of the direct applications of modern diagnostic standards and definitions directly when re-examining old records must be recognized.
3. In the scientific world, where different ideas, hypotheses, and concepts compete for attention, independent examination of original data and documents is still a fundamental prerequisite before each scientist participates in discussion or debate on a particular subject.

**Top Five Papers**

1. Halstead SB. Reappearance of chikungunya, formerly called dengue, in the Americas. Emerg Infect Dis. 2015;21: 557–561.
2. Christie J. On epidemics of dengue fever: Their diffusion and etiology. Glasgow Med J. 1881;16: 161–176.
3. Carey DE. Chikungunya and dengue: A case of mistaken identity? J Hist Med. 1971;26: 243–262.
4. Hirsch A. Dengue. In: Handbook of Geographical and Historical Pathology. London: The New Sydenham Society; 1883: vol. 1, 55–81.
5. Royal College of Physicians, London, Joint Committee. The Nomenclature of Diseases. Dengue-definition. London: W.J. & S. Golbourn; 1869: p5.
